# Identifying diabetes cases from administrative data: a population-based validation study

**DOI:** 10.1186/s12913-018-3148-0

**Published:** 2018-05-02

**Authors:** Lorraine L. Lipscombe, Jeremiah Hwee, Lauren Webster, Baiju R. Shah, Gillian L. Booth, Karen Tu

**Affiliations:** 1 Women’s College Research Institute, Women’s College Hospital, 76 Grenville Street, Toronto, ON M5S 1B1 Canada; 2 Department of Medicine, University of Toronto, Suite RFE 3-805, 200 Elizabeth Street, Toronto, ON M5G 2C4 Canada; 3 Institute for Clinical Evaluative Sciences, G1 06, 2075 Bayview Avenue, Toronto, ON M4N 3M5 Canada; 4 Institute of Health Policy, Management and Evaluation, University of Toronto, 4th Floor, 155 College St, Toronto, ON M5T 3M6 Canada; 5 Dalla Lana School of Public Health, University of Toronto, 6th Floor, 155 College St, Toronto, ON M5T 3M7 Canada; 6 Sunnybrook Health Sciences Centre, 2075 Bayview Avenue, Toronto, ON M4N 3M5 Canada; 7 Li Ka Shing Knowledge Institute, St. Michael’s Hospital, 30 Bond St, Toronto, ON M5B 1W8 Canada; 8 Department of Community and Family Medicine, University of Toronto, 5th Floor, 500 University Avenue, Toronto, ON M5G 1V7 Canada; 9 University Health Network, R. Fraser Elliot Building, 1st Floor, 190 Elizabeth St, Toronto, ON M5G 2C4 Canada

**Keywords:** diabetes, validation methods, administrative databases, electronic medical record data

## Abstract

**Background:**

Health care data allow for the study and surveillance of chronic diseases such as diabetes. The objective of this study was to identify and validate optimal algorithms for diabetes cases within health care administrative databases for different research purposes, populations, and data sources.

**Methods:**

We linked health care administrative databases from Ontario, Canada to a reference standard of primary care electronic medical records (EMRs). We then identified and calculated the performance characteristics of multiple adult diabetes case definitions, using combinations of data sources and time windows.

**Results:**

The best algorithm to identify diabetes cases was the presence at any time of one hospitalization or physician claim for diabetes AND either one prescription for an anti-diabetic medication or one physician claim with a diabetes-specific fee code [sensitivity 84.2%, specificity 99.2%, positive predictive value (PPV) 92.5%]. Use of physician claims alone performed almost as well: three physician claims for diabetes within one year was highly specific (sensitivity 79.9%, specificity 99.1%, PPV 91.4%) and one physician claim at any time was highly sensitive (sensitivity 93.6%, specificity 91.9%, PPV 58.5%).

**Conclusions:**

This study identifies validated algorithms to capture diabetes cases within health care administrative databases for a range of purposes, populations and data availability. These findings are useful to study trends and outcomes of diabetes using routinely-collected health care data.

**Electronic supplementary material:**

The online version of this article (10.1186/s12913-018-3148-0) contains supplementary material, which is available to authorized users.

## Background

The number of people with diabetes worldwide has quadrupled in the last three decades, with a staggering 422 million individuals now affected [1]. Population-based data on diabetes trends are becoming increasingly important to assist health care planners in managing this epidemic. Health care administrative data sources are often used to identify diabetes cases, in order to determine risk factors for diabetes, to report epidemiologic trends, to track complications and outcomes within diabetes patients, and to evaluate health service utilization and gaps in quality of care. As with all data that is routinely collected for other purposes, accuracy and completeness of the information may be compromised due to under-reporting or misclassification of cases. Differing case definitions and algorithms may also limit comparisons between jurisdictions.

In Canada, the Canadian Chronic Disease Surveillance System (CCDSS) identifies and monitors individuals with diabetes and other chronic conditions using a common definition [2]. The CCDSS uses routinely-collected provincial health care administrative records to identify diabetes cases, which are defined based on 1 hospitalization or 2 physician visit claims over a two-year period bearing a diagnostic code for diabetes [3]. That definition was validated in Ontario against records from primary care charts and was found to have a sensitivity of 86%, a specificity of 97%, and a positive predictive value (PPV) of 80% [4]. This algorithm has been used extensively for diabetes research to report epidemiologic trends [5, 6], quantify risk factors [7–11], evaluate outcomes [12–15], and identify health care gaps [16–18]. However, while the specificity of this definition is high, it has been shown that even modest compromises in positive predictive value increases the risk of misclassification bias [19]. This may result in sizeable errors in disease prevalence in the context of relatively uncommon conditions and large sample sizes. For instance, the 2005 Ontario Diabetes Database was estimated to have a 3% ‘false positive’ rate and 16% ‘false negative’ rate, meaning that as many as 249,840 individuals were mislabelled as having diabetes and 93,102 persons without diabetes were missed altogether [19].

One way to address this issue is to limit case definitions for diabetes to those with high PPV. Generally a PPV of 70% or greater has been considered optimal for disease algorithms using administrative or claims data [20]. However an even higher PPV (e.g. > 80%), combined with high specificity (> 98%) may be preferable in large study samples to minimize the inclusion of false positive cases [19]. One rule may thus not be sufficient for all purposes, populations, and database settings. First, the need to prioritize specificity and PPV (to identify a diabetes cohort) versus sensitivity and negative predictive value, NPV (to exclude persons with pre-existing diabetes) may vary for different research objectives and purposes. Second, PPV is highly dependent on the prevalence of disease in a particular population necessitating unique algorithms based on underlying prevalence (e.g. young versus older) [20]. Third, algorithms need to be flexible to account for variations in data availability across settings (e.g. medication data, special fee codes).

In that context, the objectives of this study were to determine optimal algorithms to identify diabetes cases within health care administrative databases for different research purposes, populations, and data sources, using diabetes identified in primary care electronic medical records (EMRs) as the reference standard. We provide performance characteristics (sensitivity, specificity, positive and negative predictive value) and probabilities of having diabetes with and without each algorithm [21].

## Methods

### Setting

We used the Electronic Medical Record Administrative data Linked Database (EMRALD), which is a comprehensive database of EMR charts from primary care physicians who use PS Suite® EMR in Ontario, Canada that is linked to administrative health care data at the individual patient level using a unique identifier [22]. Data from EMRALD held at the Institute for Clinical Evaluative Sciences were used as the reference standard to assess the performance of administrative data to capture the presence of diabetes. Data are collected annually and on a voluntarily basis from physicians. EMRALD currently contains data for over 400 physicians and over 500,000 patients in 43 clinics distributed throughout Ontario. The volunteering physicians participate by signing data sharing agreements, which allows EMRALD to collect de-identified individual patient level information without patient consent because of the prescribed entity status of ICES. EMRALD collects all of the EMR patient records of the participating physicians, which includes the cumulative patient profile (problem list, past medical history, family history, risk factors, allergies, immunizations and current treatments), laboratory test results, prescriptions, specialist consultation letters, discharge summaries and diagnostic tests for all clinical encounters. Patients and physicians are not contacted or interviewed for data collection purposes. The comprehensiveness of the data has been evaluated and all data go through data quality checks after collection and before research use [22, 23].

### Reference standard cohort

EMRALD data from 296 physicians and 258,760 rostered patients were collected between April and July 2013. To be included in this study, physicians had to be using the EMR for at least 1 year. Patients were included if they were 20 years of age or older as of March 31, 2011, had an EMR for at least 1 year, and were active within their physician’s practice (i.e. at least one visit within 3 years) since data collection. This ensured physicians had reasonable time to populate the EMRs with the patients’ full medical history and profile. Patients were identified as having diabetes if diabetes or one of its synonyms were listed in the cumulative patient profile, or they had any of the following: haemoglobin A1c greater than 7%, two abnormal blood glucose tests [fasting blood sugar(s) greater than or equal to 7.0 mmol/L, or a random blood sugar(s) greater than or equal to 11.1 mmol/L], or a prescription for an anti-diabetic medication (insulin or an oral hypoglycemic agent) unless the record reported metformin for pre-diabetes or polycystic ovarian disease. Patients with impaired glucose tolerance, impaired fasting glucose, or gestational diabetes were excluded from the case definition. This case finding method searches the EMR’s structured data using the case definition defined above, and has been previously validated using manual chart abstraction [24, 25], with a sensitivity of 90.9%, specificity of 99.2%, and positive predictive value of 94.9%.

### Administrative databases

We tested algorithms to identify diabetes within the following administrative databases. We used the discharge abstracts prepared by the Canadian Institute for Health Information (CIHI) to identify patients who were admitted to hospitals with a diagnosis of diabetes in any of the diagnosis fields (ICD-9 code 250; ICD-10 codes E10, E11, E13, E14) available from 1988 onwards. We used the Ontario Health Insurance Plan (OHIP) database, which captures all physician services claims, to identify claims with a diagnosis of diabetes (ICD-9 code 250) available from 1991, and with diabetes-specific fee codes that are used by Ontario primary care physicians for diabetes care (K030 since 2002; K045 since 2010, K046 since 2011, and Q040 since 2006). To capture medication data, we used the Ontario Drug Benefit database, which records prescriptions for medications covered by this plan for all Ontarians aged 65 years or older and those on social assistance available from 1991. The Registered Persons database was used to collect demographic information including age and sex. All relevant records from these data sources covered fiscal years 1991 (April 1 to March 31) to 2013. All administrative and EMR data were linked through a reproducibly scrambled unique health care identifier. Patients included in the EMRALD reference standard cohort that could not be linked to administrative databases were excluded.

### Administrative data algorithms

We tested various algorithms to identify diabetes cases through combinations of records from physician claims and hospital discharge abstracts, based on the presence of a diabetes diagnosis and/or prescription claims for anti-hyperglycemic medications. We specifically tested the performance characteristics of the standard CCDSS algorithm, which defines a diabetes case based on 2 physician claims or 1 hospitalization in a 2-year period bearing a diagnosis of diabetes^4^. Additional algorithms varied based on the number of physician service claims needed (1, 2, or 3 claims, +/− the presence of diabetes-specific fee codes), the data sources used (hospital +/− same-day surgery records) and the time window in which case definitions needed to be met (1,2, or 3 year periods). Diabetes-specific fee codes are used by primary care providers in Ontario to submit claims for comprehensive diabetes care, which are exclusively used for diabetes patients.

### Analysis

Diabetes prevalence estimates were calculated using the reference standard and the specified algorithm. Sensitivity and specificity were calculated as the proportion of diabetes cases identified by tested algorithms with and without diabetes according to the EMR reference standard. Positive predictive value (PPV), negative predictive value (NPV), and kappa were also calculated. We also estimated probabilities of having diabetes with (positive) and without (negative) meeting the case definition for each algorithm. Probabilities were calculated using Bayesian analyses, based on pre-test probabilities (prevalence) and likelihood ratios^21^. Ninety-five percent confidence intervals (95% CI) for proportions were calculated using binomial approximation methods. We sought algorithms that maximized sensitivity and PPV, while having the shortest time frame for case definition.

For our primary analysis, we used all available years of data to define diabetes cases within administrative databases (‘ever look-back period’, 1991–2013). To determine the performance of algorithms that are annually updated, as a secondary analysis we limited case definitions to data available in the most recent year before the reference standard (‘1 year look-back period’, 2012 to 2013). Analyses were stratified to assess potential differences by sex and by age (20–40, 41–64, and ≥ 65 years). All analyses were performed using SAS version 9.3 (SAS Institute, Cary, NC, USA).

## Results

**Fig. 1:**
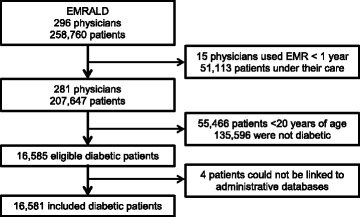
Flow diagram of patients with diabetes from primary care electronic medical records

There were 296 physicians and 258,760 rostered patients under their care. Fifteen physicians were excluded because they had not been using the EMR for at least one year, including 3248 patients under their care. Of the remaining patients, 103,331 patients were < 20 years of age and had an electronic medical record (EMR) for at least 1 year, leaving a reference cohort of 152,177 patients. Within this cohort, 16,581 (10.9%) patients had diabetes based on the validated definition and there were 135,596 persons without diabetes (Fig. 1). The mean age of diabetes patients was 62.9 (standard deviation, SD 13.6) years and 53.5% were male. The mean age of those without diabetes was 49.0 (SD 16.7) years and 40.8% were male.

### Algorithms using all available data (‘ever look-back period’, 1991–2013)

For the primary analysis, we tested administrative data algorithms against the EMR reference standard for which case definitions could be met *at any time (1991–2013)* prior to the EMR record (2012–2013). Relevant algorithms are presented in Table 1. When all available data were considered, the algorithm with the best specificity and PPV while maintaining sensitivity above 80% was either 1 hospitalization *or* 1 physician claim *and* either 1 prescription for an anti-hyperglycemic drug *or* 1 diabetes-specific fee code at any time (sensitivity 84.2%, specificity 99.2%, PPV 92.5%). Using this algorithm, the positive probability of diabetes was 93.6% (probability that an individual meeting this definition has diabetes) and the negative probability was 2.17% (probability that an individual not meeting this definition has diabetes). Omitting prescriptions from that rule reduced sensitivity to 77.2%, and omitting diabetes fee codes reduced sensitivity to 50.2% (Table 1). The algorithms with the highest sensitivity were 1 diabetes physician claim or 1 prescription at any time (94.4%), but had moderate specificity (91.8%) and PPV (58.4%). The algorithm with the highest sensitivity (88.6%) while maintaining specificity above 98% and PPV above 80% was 2 physician claims in 1 year *or* 1 prescription and 1 physician claim at any time (positive probability 86.1%, negative probability 1.6%).

**Table 1:** Validation of administrative data algorithms to adults identified with diabetes using clinical data from primary care electronic medical records as a reference standard; all adults (prevalence 10.9%), using all administrative data available from 1991 to 2013

Algorithm	Sensitivity (%) (95% CI)	Specificity (%) (95% CI)	PPV (%) (95% CI)	NPV (%) (95% CI)	Prevalence	Kappa	Probability+	Probability-
Physician claims only								
1 P	93.6(93.3–94.0)	91.9(91.7–92.0)	58.5(57.9–59.1)	99.2(99.1–99.2)	17.5%	0.68	61.7%	0.96%
2 P in 1 yr	87.2(86.7–87.7)	98.1(98.0–98.1)	84.6(84.1–85.2)	98.4(98.4–98.5)	11.2%	0.84	86.5%	1.79%
3 P in 1 yr	79.9(79.3–80.6)	99.1(99.0–99.1)	91.4(91.0–91.9)	97.6(97.5–97.7)	9.5%	0.84	92.5%	2.75%
2 P in 2 yr	88.4(87.9–88.9)	97.8(97.8–97.9)	83.4(82.9–84.0)	98.6(98.5–98.6)	11.6%	0.84	84.9%	1.63%
3 P in 2 yr	83.1(82.5–83.7)	98.9(98.8–98.9)	90.1(89.7–90.6)	98.0(97.9–98.0)	10.1%	0.85	91.3%	2.33%
Inclusion of prescription claims								
1 Rx	50.7(50.0–51.5)	99.8(99.8–99.9)	97.3(97.0–97.7)	94.3(94.2–94.4)	5.7%	0.64	97.2%	6.44%
1 P or 1 Rx	94.4(94.0–94.7)	91.8(91.6–91.9)	58.4(57.8–59.0)	99.3(99.2–99.3)	17.6%	0.68	61.6%	0.84%
1 P and 1 Rx	50.0(49.3–50.8)	99.9(99.9–99.9)	98.5(98.3–98.8)	94.2(94.1–94.4)	5.5%	0.64	98.6%	6.52%
(2 P in 1 yr) or (1Rx and 1 P)	88.6(88.1–89.1)	98.0(98.0–98.1)	84.6(84.1–85.1)	98.6(98.5–98.7)	11.4%	0.85	86.1%	1.60%
Inclusion of hospital records								
H	36.7(36.0–37.5)	99.6(99.6–99.6)	91.9(91.2–92.5)	92.8(92.7–92.9)	4.4%	0.49	92.7%	8.14%
H or 1 P	94.0(93.6–94.3)	91.7(91.5–91.8)	58.0(57.4–58.6)	99.2(99.2–99.2)	17.7%	0.67	61.2%	0.90%
H or 1 Rx	61.3(60.5–62.0)	99.5(99.4–99.5)	93.3(92.8–93.7)	95.5(95.3–95.6)	7.2%	0.72	94.5%	5.14%
H or (2 P in 1 yr)	88.4(87.9–88.8)	97.8(97.7–97.9)	83.0(82.4–83.5)	98.6(98.5–98.6)	11.6%	0.84	84.9%	1.63%
H or (3 P in 1 yr)	82.4(81.8–83.0)	98.8(98.7–98.8)	89.1(88.6–89.6)	97.9(97.8–97.9)	10.1%	0.84	90.5%	2.42%
H or (2 P in 2 yr) ^a^	89.3(88.9–89.8)	97.6(97.5–97.7)	81.9(81.3–82.4)	98.7(98.6–98.7)	11.9%	0.84	83.8%	1.51%
H or (3 P in 2 yr)	84.9(84.4–85.5)	98.6(98.5–98.6)	88.0(87.5–88.5)	98.2(98.1–98.2)	10.5%	0.85	89.4%	2.09%
Physician claims, hospital records, and prescription claims								
(H or (2 P in 1 yr)) or 1 Rx	90.0(89.5–90.4)	97.7(97.6–97.8)	82.6(82.0–83.1)	98.8(98.7–98.8)	11.9%	0.84	84.5%	1.41%
(H or (2 P in 2 yr)) or 1 Rx	90.7(90.3–91.2)	97.5(97.4–97.6)	81.5(80.9–82.0)	98.9(98.8–98.9)	12.1%	0.84	83.5%	1.31%
(H or (3 P in 2 yr)) or 1 Rx	87.4(86.9–87.9)	98.5(98.4–98.5)	87.5(87.0–88.0)	98.5(98.4–98.5)	10.9%	0.86	89.0%	1.75%
(H or 1 P) and 1 F	77.2(76.5–77.8)	99.2(99.2–99.3)	92.6(92.1–93.0)	97.3(97.2–97.3)	9.1%	0.82	93.1%	3.10%
(H or 1 P) and 1 Rx	50.2(49.4–50.9)	99.9(99.9–99.9)	98.5(98.2–98.7)	94.2(94.1–94.4)	5.6%	0.64	98.6%	6.50%
(H or 1 P) and (1 Rx or 1 F)	84.2(83.6–84.7)	99.2(99.1–99.2)	92.5(92.1–93.0)	98.1(98.0–98.2)	9.9%	0.87	93.6%	2.17%

*PPV* positive predictive value, *NPV* negative predictive value; probability+, probability of having diabetes with the algorithm, *probability-* probability of having disease without the algorithm, *H* hospital discharge abstracts bearing a diagnosis of diabetes from the Canadian Institute for Health Information Discharge Abstract Database, *P* physician claims for a diabetes diagnosis (ICD-9 250) from the Ontario Health Insurance Plan Physician Claims Database, *Rx* prescription for an anti-hyperglycemic medication from the Ontario Drug Benefit Database, *F* diabetes specific physician fee code from the Ontario Health Insurance Plan Physician Claims Database, *yr* year

^a^ *Current CDSS algorithm*

Reference standard: EMR chart – adult (≥20 years old) with diabetes or one of its synonyms were listed in the cumulative patient profile or they had any of the following: haemoglobin A1c greater than 7%, two abnormal blood glucose tests [fasting blood sugar(s) greater than or equal to 7.0 mmol/L, or a random blood sugar(s) greater than or equal to 11.1 mmol/L], or a prescription for an anti-hyperglycemic medication (insulin or an oral hypoglycemic agent). Patients were excluded if they only had a record of gestational diabetes

We also evaluated rules using only hospitalizations or physician claims (Table 1). If physician claims alone are used, 3 diabetes physician claims in 1 year had the best specificity (99.1%) and PPV (91.4%) but a sensitivity of 79.9%. Using only 2 claims in 1 year increased sensitivity to 87.2% but reduced PPV to 84.6%, and expanding to 3 claims over 2 years increased sensitivity to 83.1% while maintaining a high PPV (90.1%). Of note, 2 physician claims in 1 year achieved comparable performance characteristics as the more complex algorithm identified above. One physician claim was highly sensitive (93.6%), but had moderate specificity (91.9%) and PPV (58.5%). One hospitalization alone was highly specific (99.6%) with a high PPV (91.9%); however sensitivity was only 36.7% (negative probability 8.1%).

Combining hospitalizations or physician claims reduced specificity and PPV in all cases, with only modest increases in sensitivity. The CCDSS algorithm of 1 hospitalization or 2 physician claims in 2 years was associated with a sensitivity of 89.3%, a specificity of 97.6% and a PPV of 81.9%. In contrast, 2 physician claims in 2 years alone had a higher PPV of 83.4%, comparable specificity (97.8%) and sensitivity was only reduced to 88.4%. Modifying the CCDSS algorithm to a hospitalization or 2 physician claims in 1 year also had better PPV with similar sensitivity. Adding prescription data to the CCDSS algorithm had negligible effects on performance, and adding physician claims with diabetes-specific fee codes increased sensitivity to 91.0%, but reduced specificity and PPV to 97.1% and 79.1% respectively (Table 1).

Diabetes prevalence using the optimal algorithm was 9.9%, compared to the reference standard of 10.9% patients with diabetes among all eligible patients in the EMR sample. In contrast, the CCDSS algorithm overestimated the prevalence at 11.9%. The algorithm that most closely matched the sample prevalence was either 1 hospitalization or 3 physician claims in 2 years, or 1 prescription alone for an anti-hyperglycemic drug (10.9%). Optimal algorithms all showed good agreement between data sources with kappa values above 0.8 (Table 1).

Performance characteristics of these algorithms did not vary by sex (data not shown), but varied by age group (Additional file 1 eTable S1) largely due to differences in diabetes prevalence and data availability (i.e. prescription data are available universally only for persons aged ≥65 years). As the sample prevalence was only 2.13% in persons aged 20 to 40 years, the sensitivity and PPV of any given algorithm were reduced while specificity was increased. For this age group, the highest PPV (81.1%) with reasonable sensitivity (77.8%) was 3 physician claims in 2 years. Two physician claims in 1 year provided higher sensitivity (81.4%) while maintaining a PPV of 70.4%. In persons aged 41–64 years, 3 physician claims in 2 years was also associated with optimal sensitivity (81.1%) and PPV (90.6%) while 2 physician claims in 1 or 2 years increased sensitivity while maintaining a PPV above 80%. In the age 65+ group, due to the high diabetes prevalence (23.5%) most algorithms were associated with higher PPV and sensitivity but lower specificity than other age groups. As in the overall population, the best algorithm for age 65+ years was 1 hospitalization or physician claim *and* 1 prescription or diabetes-specific fee code (sensitivity 90.7%, PPV 92.6). Using physician claims alone, 3 claims in 2 years performed best. Of note, 1 prescription for an anti-hyperglycemic drug at any time was associated with a sensitivity of 77.1%, specificity of 99.5%, and a PPV of 98.1% in persons aged 65 or older.

### Algorithms using most recent year of data (‘1 year look-back period’, 2012–2013)

We then tested algorithms using only the most recent year of administrative data prior to the reference standard diagnosis (‘1-year look-back’ period, 2012–2013). This approach resulted in much lower sensitivity but higher specificity for all algorithms compared to the use of all available data (Table 2). One hospitalization or 1 physician claim for diabetes within the previous year had a sensitivity of 77.9%, specificity of 99.2%, and a PPV of 92.3%. The best sensitivity was achieved with 1 physician claim or 1 prescription for an anti-hyperglycemic medication within the previous year, with a sensitivity of 82.6%, specificity of 99.2%, and PPV of 98.5%. Therefore using a 1-year lookback period provides good positive probability but negative probability remains modest for most algorithms.

**Table 2:** Validation of administrative data algorithms to identify adults identified with diabetes using clinical data from primary care electronic medical records as a reference standard; all adults, using the most recent year of administrative data (2012–2013)

Algorithm	Sensitivity (%) (95% CI)	Specificity (%) (95% CI)	PPV (%) (95% CI)	NPV (%) (95% CI)	Prevalence	Kappa	Probability+	Probability-
Physician claims only								
1 P	76.4(75.8–77.1)	99.2(99.2–99.3)	92.3(91.9–92.7)	97.2(97.1–97.3)	9.01%	0.82	92.1%	3.21%
2 P in 1 yr	58.5(57.8–59.3)	99.8(99.8–99.8)	97.4(97.1–97.7)	95.2(95.1–95.3)	6.54%	0.71	97.3%	5.48%
3 P in 1 yr	41.2(40.5–42.0)	99.9(99.9–99.9)	98.3(98.0–98.6)	93.3(93.2–93.4)	4.57%	0.55	98.1%	7.58%
Inclusion of prescription claims								
1 Rx	45.9(45.2–46.7)	100.0(100.0–100.0)	99.3(99.1–99.5)	93.8(93.7–93.9)	5.03%	0.60	–	7.01%
1 P or 1 Rx	82.6(82.1–83.2)	99.2(99.1–99.2)	92.6(92.2–93.0)	97.9(97.8–98.0)	9.72%	0.86	92.7%	2.39%
1 P and 1 Rx	39.7(39.0–40.5)	100.0(100.0–100.0)	99.8(99.7–99.9)	93.1(93.0–93.3)	4.33%	0.54	–	7.75%
(2 P in 1 yr) or (1Rx and 1 P)	65.9(65.1–66.6)	99.8(99.8–99.8)	97.6(97.4–97.9)	96.0(95.9–96.1)	7.35%	0.77	97.6%	4.55%
Inclusion of hospital records								
H	9.8(9.4–10.3)	99.9(99.9–100.0)	95.6(94.6–96.6)	90.1(89.9–90.2)	1.12%	0.16	92.3%	11.18%
H or 1 P	77.9(77.2–78.5)	99.2(99.1–99.2)	92.0(91.6–92.5)	97.3(97.3–97.4)	9.21%	0.83	92.3%	3.01%
H or 1 Rx	48.6(47.9–49.4)	99.9(99.9–99.9)	98.5(98.2–98.8)	94.1(94.0–94.2)	5.38%	0.62	98.3%	6.69%
H or (2 P in 1 yr)	61.6(60.9–62.4)	99.8(99.7–99.8)	96.9(96.6–97.3)	95.5(95.4–95.6)	6.92%	0.73	97.4%	5.09%
H or (3 P in 1 yr)	45.7(44.9–46.4)	99.9(99.8–99.9)	97.6(97.2–97.9)	93.8(93.6–93.9)	5.10%	0.59	98.2%	7.04%
Physician claims, hospital records, prescription claims								
(H or (2 P in 1 yr)) or 1 Rx	73.1(72.5–73.8)	99.7(99.7–99.8)	97.1(96.8–97.4)	96.8(96.7–96.9)	8.21%	0.82	96.8%	3.62%
(H or 1 P) and (1 Rx or 1 F)	67.3(66.6–68.0)	99.8(99.7–99.8)	97.3(97.0–97.6)	96.1(96.0–96.2)	7.53%	0.78	97.6%	4.37%
(H or 1 P) and 1 F	56.4(55.7–57.2)	99.8(99.8–99.8)	96.9(96.5–97.2)	94.9(94.8–95.0)	6.34%	0.69	97.2%	5.74%
(H or 1 P) and 1 Rx	40.6(39.9–41.4)	100.0(100.0–100.0)	99.8(99.7–99.9)	93.2(93.1–93.4)	4.43%	0.55	–	7.65%

*PPV* positive predictive value, *NPV* negative predictive value, *H* hospital discharge abstracts bearing a diagnosis of diabetes from the Canadian Institute for Health Information Discharge Abstract Database, *P* physician claims for a diabetes diagnosis (ICD-92 50) from the Ontario Health Insurance Plan Physician Claims Database, *Rx* prescription for an anti-hyperglycemic medication from the Ontario Drug Benefit Database, *F*, diabetes specific physician feecode from the Ontario Health Insurance Plan Physician Claims Database, *S* same day surgery admission bearing a diagnosis of diabetes from the Canadian Institute for Health Information Discharge Abstract Database; yr., year

Reference standard: EMR chart – adult (≥20 years old) with diabetes or one of its synonyms were listed in the cumulative patient profile or they had any of the following: haemoglobin A1c greater than 7%, two abnormal blood glucose tests [fasting blood sugar(s) greater than or equal to 7.0 mmol/L, or a random blood sugar(s) greater than or equal to 11.1 mmol/L], or a prescription for an anti-hyperglycemic medication (insulin or an oral hypoglycemic agent). Patients were excluded if they only had a record of gestational diabetes

## Discussion

This study used linked EMR data to provide a set of validated algorithms to identify diabetes cases within health care administrative databases. The algorithm with the best performance characteristics used linked data from hospitalization, physician claim, and prescription databases. A combination of one hospitalization or physician claim for a diabetes diagnosis AND one prescription for an anti-diabetic medication or 1 physician claim with a diabetes-specific fee code at any time was associated with a positive predictive value of 93%, a specificity of 99%, and a sensitivity of 84%. These findings suggest that health care administrative data can accurately capture the majority of diabetes patients receiving care within the health care system. While this algorithm demonstrated good performance, it requires linkage of physician claims to prescription data and incorporates a diabetes-specific diabetes fee code only available in the province of Ontario limiting its utility to the Ontario setting. However, we found that algorithms relying on physician claims for diabetes alone performed almost as well thus making them applicable to other health care settings.

Previous validation studies identified the algorithm of 1 hospitalization or 2 physician claims in any 2-year period as having the best performance characteristics [3, 4]. This algorithm has been widely used by the CCDSS and Canadian researchers to study the trends, care, and outcomes of diabetes. Our study expanded on those findings to test a larger number of algorithms. Reassuringly, we confirmed that this algorithm continues to perform well: it was associated with 89% sensitivity, 98% specificity, and a positive predictive value of 82%. However, we found that the inclusion of hospitalization data only increases sensitivity by 1% and leads to a lower positive predictive value compared to physician claims alone. Simpler algorithms that used only physician claims data had comparable or better positive predictive value as the more complex CCDSS algorithm. The highest positive predictive value (91%) and specificity (99%) was achieved with 3 claims in any 2-year period, which had a sensitivity of 83%. Similarly, we found that limiting the observation period to 2 physician claims in any one-year period only drops sensitivity by 2% (87%) with maintenance of high specificity (98%) and positive predictive value (85%).. Our study therefore supports the use of more simplified algorithms over the more complex CCDSS algorithm, allowing for diabetes to be accurately identified using a single database and in a shorter time period. While all provinces in Canada have access to hospital discharge data from the Canadian Institute for Health Information (CIHI), the linkage of physician claims data to CIHI required by the CCDSS algorithm may be more limited in some provinces. The algorithms that use physician claims alone could therefore be widely applied across Canadian provinces. For studies requiring high specificity, using 2 (sensitivity 87%, specificity 98%) or 3 (sensitivity 80%, specificity 99%) physician claims for diabetes in a one-year period would perform best. Conversely for studies requiring high sensitivity (e.g. to exclude diabetes cases), diabetes could be defined using 1 physician claim for diabetes at any time (sensitivity 94%, specificity 92%).

The performance of algorithms varied by age group, largely due to differences in prevalence of diabetes. In general, a given algorithm had lower sensitivity and positive predictive value but higher specificity in younger versus older age groups, due to their lower prevalence of diabetes. Age-specific performance characteristics therefore need to be taken into consideration in diabetes studies that restrict or stratify cohorts by age group. The optimal algorithm for individuals aged 20 to 40 years was 3 physician claims in 2 years, which is comparable to the validated algorithm for pediatric cases of diabetes (4 physician claims in 2 years) [26]. Of note, because prescription medications are captured for all individuals aged 65 years or older in Ontario databases, use of any prescription record for an anti-diabetes medication alone was associated with a positive predictive value of 98% and specificity of 99.5% in that age group. These findings provide support for use of prescription data alone to identify patients with diabetes in databases where all medications are captured, such as in the Canadian provinces of British Columbia, Quebec, Saskatchewan, and Manitoba. However, as sensitivity was only 77%, use of prescription claims should be limited to conditions whereby specificity is more important than sensitivity.

Previous studies did not clarify the optimal length of look-back period needed to capture or exclude baseline cases of diabetes in health care databases. We showed that a one-year look-back period is sufficient to accurately capture diabetes cases with a high positive predictive value, but sensitivity is reduced leading to a greater proportion of missed cases. Use of 1 physician claim or 1 anti-diabetic prescription in the previous year, however, was able to identify 83% of cases with a positive predictive value of almost 99%. The advantage of having a one year look-back period allows for reporting up to the most recent year available data. Requiring a longer look-back period to meet a case definition necessitates a wider time-frame prior to cohort entry, which may compromise the eligibility of study participants and follow-up time. Although we lost sensitivity with a one year look back period, the substantial increase in positive predictive value to near optimal levels suggests a low number of false positives. Therefore the annual application of the algorithm would be an accurate and conservative approach that could help reduce the accumulation of false positives.

Strengths of this study include the use of a large population-based sample, completeness of data capture in a single-payer health care system, use of a manually validated reference standard, and testing of a large number of algorithms. We also had access to EMRs linked to administrative data, which allowed for efficient testing of multiple algorithms that would not be feasible with manual chart review [27]. However there are limitations to this study. First, our reference population was restricted to patients who had a primary care physician and had at least 1 visit within the previous 3 years. The superior performance of physician claim-based algorithms may be partly attributed to the dependence on primary care visits to identify our reference cases; we were not able to determine how well these algorithms would identify patients not actively managed by a primary care physician. This reference standard does not capture diabetes cases uniquely identified in a hospital setting; therefore our study may have underestimated the potential increase in sensitivity achieved with adding hospitalization data to physician claims. However given the nature of the disease, it is unlikely that patients with diabetes would not see their family physician at least once in a 3-year period. We also did not have data on undiagnosed diabetes or on persons who do not regularly access the health care system. Second, as we did not have data on laboratory tests in our administrative data, we could not test algorithms that incorporate blood tests such as glucose or HbA1c. Third, while we found that prescription data alone performed well in persons aged 65 years or older, we could not determine whether this would be generalizable to younger age groups if medication data were available. Finally, our study was limited to the Ontario health care context and results may not be applicable to other settings.

## Conclusion

In conclusion, we identified performance characteristics for a set of algorithms that can be used to accurately capture diabetes cases within health care administrative databases. We provide optimal algorithms overall and by age group, and using both linked and unlinked data from different databases. These findings will be useful for researchers and policymakers seeking to study trends and outcomes of diabetes within a Canadian context, and may also be applicable to other settings with similar data.

## Additional file

Additional file 1: Validation of administrative data algorithms to identify adult patients who were identified with diabetes using clinical data from primary care electronic medical records as a reference standard by age group, using all administrative data available from 1991-2013. (DOCX 22 kb)

## Abbreviations

CCDDS: Canadian Chronic Disease Surveillance System
CIHI: Canadian Institute for Health Information
EMR: Electronic Medical Records
EMRALD: Electronic Medical Record Administrative data Linked Database
NPV: Negative Predictive Value
OHIP: Ontario Health Insurance Plan
PPV: Positive Predictive Value
