# Supplementary material for: Identifying diabetes cases from administrative data: a population-based validation study
Source: BMC Health Serv Res. 2018 May 2;18:316. doi: 10.1186/s12913-018-3148-0 (PMC5932874; doi:10.1186/s12913-018-3148-0)
Supplement: Supplementary file 1 — Validation of administrative data algorithms to identify adult patients who were identified with diabetes using clinical data from primary care electronic medical records as a reference standard by age group, using all administrative data available from 1991-2013. (DOCX 22 kb) [file 12913_2018_3148_MOESM1_ESM.docx]

| Algorithm | Age 20-40 years (Prevalence 2.13%) | | | | Age 41-64 years (Prevalence 11.2%) | | | | Age 65+ years (Prevalence 23.5%) | | | |
| --- | --- | --- | --- | --- | --- | --- | --- | --- | --- | --- | --- | --- |
|  | **Sn (%)**  **(95% CI)** | **Sp (%)**  **(95% CI)** | **PPV (%)**  **(95% CI)** | **NPV (%)**  **(95% CI)** | **Sn (%)**  **(95% CI)** | **Sp (%)**  **(95% CI)** | **PPV (%)**  **(95% CI)** | **NPV (%)**  **(95% CI)** | **Sn (%)**  **(95% CI)** | **Sp (%)**  **(95% CI)** | **PPV (%)**  **(95% CI)** | **NPV (%)**  **(95% CI)** |
| Physician claims only | | | | | | | | | | | | |
| 1 P | 90.0  (88.2-91.9) | 95.8  (95.6-96.0) | 31.8  (30.1-33.5) | 99.8  (99.7-99.8) | 92.9  (92.4-93.5) | 91.6  (91.4-91.8) | 58.4  (57.6-59.3) | 99.0  (99.0-99.1) | 94.9  (94.4-95.4) | 84.9  (84.4-85.3) | 65.8  (64.9-66.7) | 98.2  (98.0-98.4) |
| 2 P in 1 yr | 81.4  (79.1-83.8) | 99.3  (99.2-99.3) | 70.6  (68.0-73.2) | 99.6  (99.5-99.7) | 85.7  (84.9-86.5) | 98.1  (98.0-98.2) | 85.1  (84.3-85.8) | 98.2  (98.1-98.3) | 91.0  (90.4-91.7) | 93.7  (93.4-94.0) | 81.6  (80.7-82.4) | 97.1  (96.9-97.4) |
| 3 P in 1 yr | 75.5  (72.8-78.1) | 99.6  (99.6-99.7) | 82.2  (79.8-84.6) | 99.5  (99.4-99.5) | 77.9  (77.0-78.8) | 99.1  (99.1-99.2) | 91.9  (91.3-92.6) | 97.3  (97.1-97.4) | 85.6  (84.8-86.4) | 96.8  (96.6-97.0) | 89.1  (88.3-89.8) | 95.6  (95.4-95.9) |
| 2 P in 2 yr | 82.0  (79.7-84.4) | 99.2  (99.1-99.3) | 69.1  (66.5-71.7) | 99.6  (99.6-99.7) | 87.1  (86.3-87.8) | 97.9  (97.7-98.0) | 83.7  (82.9-84.5) | 98.4  (98.3-98.5) | 91.7  (91.1-92.3) | 93.4  (93.1-93.7) | 81.0  (80.2-81.9) | 97.4  (97.2-97.6) |
| 3 P in 2 yr | 77.8  (75.2-80.3) | 99.6  (99.5-99.7) | 81.1  (78.7-83.6) | 99.5  (99.5-99.6) | 81.1  (80.3-82.0) | 98.9  (98.9-99.0) | 90.6  (90.0-91.3) | 97.6  (97.5-97.8) | 87.7  (87.0-88.5) | 96.3  (96.1-96.6) | 88.0  (87.3-88.8) | 96.2  (96.0-96.5) |
| Inclusion of prescription claims | | | | | | | | | | | | |
| 1 Rx | 27.4  (24.7-30.2) | 99.9  (99.8-99.9) | 81.8  (77.8-85.9) | 98.4  (98.3-98.6) | 29.1  (28.1-30.1) | 99.9  (99.9-99.9) | 97.7  (97.2-98.3) | 91.8  (91.6-92.0) | 77.1  (76.2-78.1) | 99.5  (99.5-99.6) | 98.1  (97.7-98.4) | 93.4  (93.1-93.7) |
| 1 P or 1 Rx | 90.9  (89.2-92.7) | 95.7  (95.5-95.9) | 31.5  (29.8-33.2) | 99.8  (99.8-99.8) | 93.3  (92.7-93.8) | 91.6  (91.4-91.8) | 58.4  (57.5-59.3) | 99.1  (99.0-99.2) | 96.0  (95.6-96.4) | 84.7  (84.3-85.2) | 65.8  (64.9-66.7) | 98.6  (98.4-98.7) |
| 1 P and 1 Rx | 26.6  (23.9-29.3) | 100.0  99.9-100.0) | 93.5  (90.7-96.3) | 98.4  (98.3-98.5) | 28.8  (27.8-29.8) | 99.9  (99.9-100.0) | 98.6  (98.2-99.1) | 91.7  (91.5-91.9) | 76.0  (75.1-77.0) | 99.7  (99.6-99.8) | 98.7  (98.4-99.0) | 93.1  (92.8-93.4) |
| (2 P in 1 yr) or (1Rx and 1 P) | 81.9  (79.6-84.3) | 99.2  (99.2-99.3) | 70.2  (67.6-72.8) | 99.6  (99.5-99.7) | 86.5  (85.7-87.2) | 98.1  (98.0-98.2) | 85.1  (84.3-85.8) | 98.3  (98.2-98.4) | 92.6  (92.0-93.2) | 93.6  (93.3-93.9) | 81.6  (80.8-82.4) | 97.6  (97.4-97.8) |
| Inclusion of hospital records | | | | | | | | | | | | |
| H | 31.6  (28.8-34.4) | 99.9  (99.9-100.0) | 90.6  (87.6-93.6) | 98.5  (98.4-98.6) | 28.7  (27.7-29.7) | 99.7  (99.6-99.7) | 91.9  (90.9-93.0) | 91.7  (91.5-91.9) | 46.0  (44.9-47.2) | 98.8  (98.6-98.9) | 92.0  (91.1-92.9) | 85.7  (85.3-86.1) |
| H or 1 P | 90.3  (88.5-92.1) | 95.7  (95.6-95.9) | 31.6  (29.9-33.3) | 99.8  (99.7-99.8) | 93.1  (92.6-93.7) | 91.5  (91.2-91.7) | 58.0  (57.1-58.8) | 99.1  (99.0-99.1) | 95.4  (94.9-95.8) | 84.3  (83.9-84.8) | 65.1  (64.2-65.9) | 98.3  (98.2-98.5) |
| H or 1 Rx | 44.8  (41.8-47.9) | 99.8  (99.8-99.8) | 83.0  (79.9-86.1) | 98.8  (98.7-98.9) | 44.6  (43.5-45.7) | 99.6  (99.6-99.7) | 93.6  (92.9-94.4) | 93.4  (93.2-93.6) | 81.4  (80.6-82.3) | 98.4  (98.2-98.5) | 93.9  (93.4-94.5) | 94.5  (94.3-94.8) |
| H or (2 P in 1 yr) | 82.0  (79.7-84.4) | 99.2  (99.1-99.3) | 69.3  (66.7-71.9) | 99.6  (99.6-99.7) | 86.6  (85.8-87.3) | 97.9  (97.8-98.0) | 83.7  (82.9-84.5) | 98.3  (98.2-98.4) | 92.1  (91.5-92.7) | 92.9  (92.6-93.2) | 79.9  (79.1-80.8) | 97.5  (97.3-97.7) |
| H or (3 P in 1 yr) | 76.5  (73.9-79.1) | 99.6  (99.5-99.6) | 80.1  (77.6-82.6) | 99.5  (99.4-99.6) | 79.8  (78.9-80.6) | 98.9  (98.8-99.0) | 90.  (89.3-90.7) | 97.5  (97.4-97.6) | 88.1  (87.4-88.8) | 95.9  (95.6-96.1) | 86.7  (85.9-87.5) | 96.3  (96.1-96.6) |
| H or (2 P in 2 yr) | 82.6  (80.3-84.9) | 99.1  (99.1-99.2) | 67.9  (65.3-70.4) | 99.6  (99.6-99.7) | 87.8  (87.0-88.5) | 97.6  (97.5-97.8) | 82.5  (81.7-83.3) | 98.4  (98.3-98.5) | 92.6  (92.1-93.2) | 92.7  (92.3-93.0) | 79.4  (78.6-80.3) | 97.6  (97.4-97.8) |
| H or (3 P in 2 yr) | 78.7  (76.3-81.2) | 99.5  (99.5-99.6) | 79.1  (76.6-81.6) | 99.5  (99.5-99.6) | 82.6  (81.8-83.4) | 98.7  (98.6-98.8) | 88.9  (88.1-89.6) | 97.8  (97.7-97.9) | 89.6  (88.9-90.3) | 95.5  (95.2-95.7) | 85.8  (85.0-86.6) | 96.8  (96.5-97.0) |
| Physician claims, hospital records, and prescription claims | | | | | | | | | | | | |
| (H or (2 P in 1 yr)) or 1 Rx | 83.3  (81.0-85.6) | 99.1  (99.0-99.2) | 66.8  (64.2-69.3) | 99.6  (99.6-99.7) | 87.5  (86.8-88.2) | 97.8  (97.7-97.9) | 83.6  (82.8-84.4) | 98.4  (98.3-98.5) | 94.2  (93.6-94.7) | 92.7  (92.4-93.0) | 79.8  (78.9-80.6) | 98.1  (97.9-98.3) |
| (H or (2 P in 2 yr)) or 1 Rx | 83.9  (81.6-86.1) | 99.0  (98.9-99.1) | 65.5  (62.9-68.0) | 99.6  (99.6-99.7) | 88.5  (87.8-89.2) | 97.6  (97.5-97.7) | 82.3  (81.5-83.1) | 98.5  (98.4-98.6) | 94.5  (94.0-95.0) | 92.4  (92.1-92.7) | 79.2  (78.4-80.1) | 98.2  (98.0-98.4) |
| (H or (3 P in 2 yr)) or 1 Rx | 80.4  (78.0-82.8) | 99.4  (99.4-99.5) | 75.6  (73.1-78.2) | 99.6  (99.5-99.6) | 84.0  (83.2-84.8) | 98.6  (98.6-98.7) | 88.7  (88.0-89.4) | 98.0  (97.9-98.1) | 92.7  (92.1-93.3) | 95.2  (94.9-95.5) | 85.6  (84.8-86.3) | 97.7  (97.5-97.9) |
| (H or 1 P) and 1 F | 67.0  (64.1-69.8) | 99.8  (99.7-99.8) | 87.1  (84.7-89.4) | 99.3  (99.2-99.4) | 75.6  (74.6-76.5) | 99.3  (99.3-99.4) | 93.3  (92.7-93.9) | 97.0  (96.9-97.1) | 82.1  (81.2-83.0) | 97.8  (97.6-98.0) | 92.0  (91.4-92.7) | 94.7  (94.4-95.0) |
| (H or 1 P) and  1 Rx | 26.7  (24.0-29.4) | 100.0  (99.9-100.0) | 93.6  (90.8-96.4) | 98.4  (98.3-98.5) | 28.8  (27.8-29.8) | 99.9  (99.9-100.0) | 98.5  (98.0-99.0) | 91.7  (91.5-91.9) | 76.3  (75.3-77.3) | 99.7  (99.6-99.8) | 98.7  (98.4-99.0) | 93.2  (92.9-93.5) |
| (H or 1 P) and  (1 Rx or 1 F) | 72.0  (69.2-74.7) | 99.8  (99.7-99.8) | 86.3  (84.0-88.6) | 99.4  (99.3-99.5) | 79.7  (78.8-80.5) | 99.3  (99.2-99.3) | 93.3  (92.7-93.9) | 97.5  (97.4-97.6) | 91.0  (90.3-91.6) | 97.6  (97.4-97.8) | 92.1  (91.5-92.7) | 97.2  (97.0-97.5) |

e-Table: Validation of administrative data algorithms to identify adult patients who were identified with diabetes using clinical data from primary care electronic medical records as a reference standard **by age group**, using **all** administrative data available from 1991-2013.
